# Taxonomic and Functional Characterization of the Microbial Community During Spontaneous *in vitro* Fermentation of Riesling Must

**DOI:** 10.3389/fmicb.2019.00697

**Published:** 2019-04-09

**Authors:** Kimmo Sirén, Sarah Siu Tze Mak, Chrats Melkonian, Christian Carøe, Jan Hendrik Swiegers, Douwe Molenaar, Ulrich Fischer, M. Thomas P. Gilbert

**Affiliations:** ^1^Institute for Viticulture and Oenology, Dienstleistungszentrum Ländlicher Raum Rheinpfalz, Neustadt an der Weinstraße, Germany; ^2^Department of Chemistry, University of Kaiserslautern, Kaiserslautern, Germany; ^3^Section for Evolutionary Genomics, Natural History Museum of Denmark, University of Copenhagen, Copenhagen, Denmark; ^4^Systems Bioinformatics, Faculty of Science, Vrije Universiteit Amsterdam, Amsterdam, Netherlands; ^5^Chr. Hansen A/S, Hoersholm, Denmark; ^6^University Museum, Norwegian University of Science and Technology, Trondheim, Norway

**Keywords:** shotgun sequencing, metabarcoding, wine, microbial diversity, alcoholic fermentation

## Abstract

Although there is an extensive tradition of research into the microbes that underlie the winemaking process, much remains to be learnt. We combined the high-throughput sequencing (HTS) tools of metabarcoding and metagenomics, to characterize how microbial communities of Riesling musts sampled at four different vineyards, and their subsequent spontaneously fermented derivatives, vary. We specifically explored community variation relating to three points: (i) how microbial communities vary by vineyard; (ii) how community biodiversity changes during alcoholic fermentation; and (iii) how microbial community varies between musts that successfully complete alcoholic fermentation and those that become ‘stuck’ in the process. Our metabarcoding data showed a general influence of microbial composition at the vineyard level. Two of the vineyards (4 and 5) had strikingly a change in the differential abundance of *Metschnikowia*. We therefore additionally performed shotgun metagenomic sequencing on a subset of the samples to provide preliminary insights into the potential relevance of this observation, and used the data to both investigate functional potential and reconstruct draft genomes (bins). At these two vineyards, we also observed an increase in non-Saccharomycetaceae fungal functions, and a decrease in bacterial functions during the early fermentation stage. The binning results yielded 11 coherent bins, with both vineyards sharing the yeast bins *Hanseniaspora* and *Saccharomyces*. Read recruitment and functional analysis of this data revealed that during fermentation, a high abundance of *Metschnikowia* might serve as a biocontrol agent against bacteria, via a putative iron depletion pathway, and this in turn could help *Saccharomyces* dominate the fermentation. During alcoholic fermentation, we observed a general decrease in biodiversity in both the metabarcoding and metagenomic data. Unexpected *Micrococcus* behavior was observed in vineyard 4 according to metagenomic analyses based on reference-based read mapping. Analysis of open reading frames using these data showed an increase of functions assigned to class Actinobacteria in the end of fermentation. Therefore, we hypothesize that bacteria might sit-and-wait until *Saccharomyces* activity slows down. Complementary approaches to annotation instead of relying a single database provide more coherent information true species. Lastly, our metabarcoding data enabled us to identify a relationship between stuck fermentations and *Starmerella* abundance. Given that robust chemical analysis indicated that although the stuck samples contained residual glucose, all fructose had been consumed, we hypothesize that this was because fructophilic *Starmerella*, rather than *Saccharomyces*, dominated these fermentations. Overall, our results showcase the different ways in which metagenomic analyses can improve our understanding of the wine alcoholic fermentation process.

## Introduction

Microbial interactions are vital to the winemaking process, with numerous different microbes known to be involved in the formation of wine flavor and aroma. Understanding this microbial diversity and their interactions throughout the process stands to enhance our knowledge of winemaking and wine complexity (Tempère et al., 2018). Although research on wine microbes has a long history (Pasteur, 1872), many significant challenges remain to be solved, not least due to difficulties in studying the composition of wine’s complex matrix. In this regard, there is considerable interest in the application of high-throughput sequencing (HTS) tools such as metabarcoding and shotgun metagenomic sequencing to wine research, given their potential to offer us more in-depth characterization of the microbial community (Belda et al., 2017; Stefanini and Cavalieri, 2018; Sirén et al., 2019).

Questions that have received considerable attention include the origin of yeasts that drive the fermentation, and how different microbes shape the fermentation (Fleet, 1990, 2003; Pretorius, 2000; Bisson et al., 2017; Varela and Borneman, 2017). However, the answers to these questions are not clear cut. For example, it remains debated as to whether sufficient *Saccharomyces cerevisiae* is present in the vineyard (thus entering the must during pressing) to drive fermentation (Martini, 1993). This question is particularly timely today, given the trend to return to spontaneous fermentation during winemaking, for reasons relating to both typicality as well as arguments that spontaneously fermented wines gain in complexity due to the more diverse microbial interactions (Di Maro et al., 2007). Furthermore, the relative importance of the vineyard versus winery flora during fermentation remains inconclusive, and little is known about how the two interact with each other. While some authors have suggested that the main contributors to fermentation originate from the vineyard flora (Bokulich et al., 2014, 2016; Morrison-Whittle and Goddard, 2018), others argue that the winery flora dominates (Stefanini et al., 2016; Ganucci et al., 2018).

A further topic of interest is the dynamics of the microbial community during the alcoholic fermentation. While alcoholic fermentation is known to result from a succession of various microbes, with *Saccharomyces* eventually dominating, details about the timing and abundances of different microbes remain of interest (Stefanini et al., 2016). It is currently understood that while microbial diversity decreases during the winemaking process (Bisson et al., 2017), some microbes can survive (Torija et al., 2001; Romano et al., 2003), such as the yeast *Metschnikowia pulcherrima* (Díaz et al., 2018) and some bacteria such as *Lactobacillus*, Lactobacillaceae, and *Gluconobacter* (Bokulich et al., 2015; Piao et al., 2015). Although traditionally how the different microbial species interact has been studied using culture-based techniques, they are increasingly targeted using culture-independent methods (Jolly et al., 2014; Bagheri et al., 2017; Sternes et al., 2017; Morgan et al., 2018; Zepeda-Mendoza et al., 2018).

Another area of growing interest relates to the role of spontaneous fermentation. Because this is driven principally by non-*Saccharomyces* yeasts, spontaneous fermentations are regarded as being able to diversify aromatic quality (Zott et al., 2011; Liu et al., 2016). However, their use remains intimidating for the industry, as they can lead to unwanted characteristics (Ciani et al., 2006), and sluggish, or even stuck, fermentations (Bisson, 1999; Bisson and Butzke, 2000). While the main reason for sluggish fermentation is often nutrient related, additional microbial interactions could play a role, for example, by reducing nutrient availability. Recently, metabarcoding data have been used to suggest that high species richness (including the presence of non-*Saccharomyces* yeasts) in must samples can negatively affect the capability of *Saccharomyces* to carry out the fermentation (Boynton and Greig, 2016). If so, the addition of sulfur (SO_2_) to the harvested grapes or must might be a means to allow desired winery microbes to dominate, by removing competition originating from unwanted vineyard microbes.

Although there is an increasing trend to apply HTS tools to study wine microbiology, with few exceptions these have been amplicon-based “metabarcoding” approaches that enable community profiling (e.g., as varied by geography, regions, *Botrytis*) as reviewed extensively by Belda et al. (2017) and Stefanini and Cavalieri (2018). Thus, there is considerable interest in profiling the whole genomic content using true metagenomic approaches, in the hope that they will also provide information about the functional pathways involved (Sirén et al., 2019). In this regard, there are currently only two shotgun sequencing-based metagenomic studies of wine fermentation published (Sternes et al., 2017; Zepeda-Mendoza et al., 2018). The first investigated the bias between metagenomics and metabarcoding in assessing community structures, while the second characterized the impact of the inoculations of *Oenococcus oeni* and *Brettanomyces bruxellensis* on volatile phenol formation.

Given the potential of HTS for investigating microbial community and interactions, and metagenomics in providing insights into genomes and genes together with their functional potential (Sirén et al., 2019), in this study, we combined metabarcoding and metagenomic sequencing in order to investigate two principal questions relating to spontaneously fermented (i.e., not deliberately inoculated) must. First, we explored how the fermentation microbial community differs as a result of vineyard, and secondly, how the microbial diversity changes during the fermentation process itself. Specifically, we chose to spontaneously ferment must samples originating from four Riesling vineyards in Pfalz (Germany). While most of the samples completed alcoholic fermentation, several exhibited sluggish fermentation. Thus, this also provided us with the opportunity to also explore the microbial basis of this phenomenon.

## Materials and Methods

**Table 1:** Summary of samples analyzed.

No.	Sample name	Metagenomic sample ID	Vineyard	Biological replicate	Sampling date	Alcohol percentage
1	w2a109		2	a	09-Oct	0.5
2	w2b109		2	b	09-Oct	1.3
3	w3a109^∗^	W1	3	a	09-Oct	1.5
4	w3b109		3	b	09-Oct	6
5	w4a109^∗^	W3	4	a	09-Oct	1
6	w4b109		4	b	09-Oct	1
7	w5a109		5	a	09-Oct	0.22
8	w5b109		5	b	09-Oct	0.14
9	w2a112		2	a	12-Oct	1.11
10	w2b112		2	b	12-Oct	1.62
11	w3a112^∗^	W2	3	a	12-Oct	5.65
12	w3b112		3	b	12-Oct	9.51
13	w4a112^∗^	W4	4	a	12-Oct	4.93
14	w4b112		4	b	12-Oct	1.79
15	w5a112^∗^	W7	5	a	12-Oct	0.87
16	w5b112^∗^	W9	5	b	12-Oct	1.49
17	w2a114		2	a	14-Oct	1.13
18	w2b114		2	b	14-Oct	1.99
19	w3a114		3	a	14-Oct	8.18
20	w3b114		3	b	14-Oct	10.64
21	w4a114^∗^	W5	4	a	14-Oct	8.44
22	w4b114		4	b	14-Oct	2.16
23	w5a114^∗^	W8	5	a	14-Oct	3.85
24	w5b114^∗^	W10	5	b	14-Oct	4.86
25	w2a120		2	a	20-Oct	1.65
26	w2b120		2	b	20-Oct	3.66
27	w3a120		3	a	20-Oct	11.41
28	w3b120		3	b	20-Oct	11.85
29	w4a120^∗^	W6	4	a	20-Oct	12.34
30	w4b120		4	b	20-Oct	3.47
31	w5a120		5	a	20-Oct	10.5
32	w5b120		5	b	20-Oct	10.34

*Each sample name can be used to identify the vineyard name (first two characters), biological replicate (a or b), and the sampling date in 2015 (last two numbers). ^∗^Samples selected for metagenomic sequencing.*

### Fermentation Set Up and Sample Collection

A fermentation experiment was carried out with grapes from four Riesling vineyards in the Pfalz wine region of Germany in Autumn 2015, in order to investigate spontaneous fermentation dynamics, i.e., fermentation derived solely from vineyard, rather than winery, microbes. From each commercial vineyard (each also has a different owner), 8 kg of grapes were handpicked following a random pattern throughout the vineyard into autoclaved sterile flat plastic bags (Neolab). These were sealed inside the vineyards and later processed at the Institute for Viticulture and Oenology, Dienstleistungszentrum Ländlicher Raum Rheinpfalz, Neustadt an der Weinstraße, Germany. The microvinifications were carried out in sterile conditions under a laminar flow hood, to restrict fermentation to only those microbes present in the vineyards. After crushing and pressing, the must from each vineyard was left in 3 L sterile autoclaved Erlenmeyer flasks (Duran, Germany) for overnight sedimentation at 4°C. After settling, must was racked into duplicate 1 L autoclaved Erlenmeyer flasks (Duran, Germany) and secured with silicone bungs attached to distilled water filled airlocks. Sample collection was done over 4 different days during alcoholic fermentation, with 4.5 mL of fermenting must collected at each timepoint for subsequent DNA analysis (total 32 extracts, Table 1), and 40 mL collected for monitoring of the fermentation by measuring various wine parameters (alcohol, density, total sugar, glucose, fructose, glycerol, titratable acidity, pH, tartaric acid, malic acid, lactic acid, citric acid, volatile acid, glycerol, yeast assimilable nitrogen, primary amino nitrogen (NOPA), and ammonium with routine FTIR analysis (WineScan FT120, FOSS Electric)]. Furthermore, the alcohol percentage was estimated by dividing the product of measured alcohol (g/mL) and sample density (g/mL) by 10 times the density of ethanol (g/mL). In general, estimation of the fermentation progress through changes in density or alcohol concentration is complicated without continual monitoring of the must. However, it has been observed that continuous monitoring can potentially expose must to contaminating microbes (Ocón et al., 2013; Pérez-Martín et al., 2014; Grangeteau et al., 2015), and therefore, we chose instead to sample four times during the fermentations. Details of samples and measured wine parameters are shown in Supplementary Table S1.

### DNA Extraction

Prior to DNA extraction, each sample, was centrifuged at 4500 × *g* for 10 min, after which the supernatant was removed and the pellet resuspended with 1 mL of ice-cold 1X PBS (pH 7.4, Life Technologies, Camarillo, CA, United States). The resuspended samples were washed twice with 1 mL of ice-cold 1X PBS to remove debris. The pellets were subsequently stored at −20°C until DNA extraction. DNA extractions were performed as described in a parallel study (Mak et al., in review), with the use of FastDNA Spin Kit for Soil (MP Biomedical, Santa Ana, CA, United States) following the manufacturer’s protocol with minor modifications. In brief, pellets were bead-beaten twice at 30 Hz for 40 s using a TissueLyser II (Qiagen, Hilden, Germany), with cooling step on ice for 2 min in between bead-beating steps. In the elution step, 105 μL of 1X TET buffer (1X TE buffer in 10 mM Tris-HCl, 1 mM EDTA, pH 8.0, Sigma–Aldrich, and 0.05% Tween 20, Sigma–Aldrich) was added to the filter column then incubated at 55°C for 5 min before elution. DNA was subsequently subjected to an extra purification step using a DNA Clean and Concentrator^TM^-5 (Zymo Research, Irvine, CA, United States), and eluted in a final volume of 55 μL of 1X TET buffer. DNA extracts were quantified using a Qubit 1.0 fluorometer with dsDNA High Sensitivity Assay kit (ThermoFisher Scientific). An extraction blank was included for every 16 samples.

### qPCR

Prior to metabarcoding PCRs, we used quantitative real-time PCR (qPCR) to both estimate the number of copies of the region, thus determine the number of PCR cycles, and to identify whether PCR inhibitors were present in the DNA extracts. For both qPCR and metabarcoding, we used fusion primers targeting the fungal internal transcribed spacer 2 region (ITS2, ITS7_F from Ihrmark et al., 2012 and ITS4_R from White et al., 1990), each containing an exclusive 8 bp multiplex identifier tag (MID tag) and MiSeq sequencing adapters.

Each qPCR reaction consisted of a 25 μL reaction volume containing 2 μL of template and 23 μL of mastermix containing 1X GeneAmp^®^10X PCR Buffer II (Applied Biosystems, United States), 2.5 mM MgCl_2_ (Applied Biosystems, United States), 0.8 mg/mL bovine serum albumin (BSA), 1 μL SYBR Green (Invitrogen, Carlsbad, CA, United States), 0.25 mM dNTPs, 0.4 μM forward primer, 0.4 μM reverse primer, 0.25 μL AmpliTaq Gold DNA polymerase (Applied Biosystems, United States), and 14.5 μL AccuGene molecular biology water (Lonza). qPCR conditions were as follows: 95°C for 5 min, followed by (95°C for 30 s, 52°C for 30 s, and 72°C for 45 s) for 45 cycles, and a final dissociation curve of 1 cycle of 95°C for 1 min, 55°C for 30 s, and 95°C for 30 s. PCRs were performed using a MX3005 qPCR machine (Agilent). Standard curves with duplicates were generated using a 10-fold serial dilution (10^1^–10^9^ copies/μL) of PCR products generated by DNA from *Cortinarius hinnuleoarmillatus* with the same primer set. Positive controls and negative controls were included.

### PCR and Metabarcode Sequencing

We applied metabarcoding to all DNA extracts. Extraction blanks, positive controls, and PCR negative controls were included to monitor for contamination. Metabarcoding PCRs were based on the same mastermix as that used in the qPCR, except replacing 1 μL SYBR Green (Invitrogen, Carlsbad, CA, United States) with 1 μL AccuGene molecular biology water (Lonza, Switzerland). PCRs were carried out in AB 2720 Thermal cycler (Applied Biosystems, United States) with the following conditions: 95°C for 5 min, followed by 33 cycles of 95°C for 30 s, 52°C for 30 s, and 72°C for 45 s, and a final elongation step of 72°C for 10 min. PCR products (∼480 bp) were visualized by electrophoresis using 2% agarose gels, then subsequently pooled together with amplicons derived from a parallel study (Mak et al., in review) into three amplicon pools. Amplicon pools were subsequently purified with QiaQuick columns (Qiagen) following the manufacturer’s protocol to remove primer dimers. An aliquot of each amplicon pool was used for quantification and size estimation using the High-Sensitivity D1000 Screen Tape for Agilent 2200 TapeStation (Agilent). Lastly, purified amplicon pools were sent for sequencing in two flow cells on the Illumina MiSeq platform in 250 bp paired-end mode at The Danish National High-Throughput DNA Sequencing Centre, Copenhagen, Denmark. This dataset comprised two-third of a MiSeq flow cell.

### Shotgun Library Construction and Metagenomics Sequencing

We additionally generated shotgun metagenomic data on a subset of ten of the samples. These were chosen as samples exhibiting normal fermentation rate, with a focus on varying alcohol levels (especially in early fermentation stages), different vineyards, and given the fungal diversity observed during the metabarcoding. Metagenomic sequencing (Table 1 and Supplementary Table S2) was performed with BGISeq technology (Fang et al., 2018), although with a customized library build. The DNA extracts were initially fragmented to around 300 bp using a Bioruptor 300 (Diagenode, Belgium) using 10 cycles of 30 s on and 90 s off. The DNA was then converted into indexed sequencing libraries using the Blunt End Multi Tubes (BEMT) protocol (Supplementary File S1), following an initial comparison of the performance of different library construction methods on DNA extracted from ferment samples (Supplementary File S1 and Supplementary Figure S5). Library blanks and index PCR blanks were included; 30 μL (input amount: <0.3–6.6 ng) of each sample was used for each library constructions (Supplementary Table S2) and 2 μL of 10 μM BGI 2.0 adapters (Supplementary Table S3) were added to each sample in the adapter-ligation step. Each library was quantified using qPCR post-construction in order to determine the appropriate number of PCR cycles to subject each to. This was done using a MX3005 qPCR machine (Agilent) with the forward primer and one indexed reverse primer. Post-qPCR, each library was subsequently PCR amplified and indexed with different indices and purified for residual adapter dimers using SPRI beads (Sigma–Aldrich) in 1.5X beads:library ratio with incubation at 37°C for 10 min and eluted in 50 μL. All libraries were then sent to BGI-Europe for sequencing, where they were pooled in equimolar concentrations for circularization, DNA nanoballs (DNBs) construction, and sequencing on the BGISeq-500 platform over four lanes in 100 bp pair-ended mode.

### Sequencing Data Analysis

#### Metabarcoding Sequencing Analysis

Metabarcoding sequence analyses were performed following the pipeline described in Feld et al. (2016), although with modifications in trimming, post-clustering, and the use of databases. Raw reads were merged and demultiplexed using vsearch v2.1.2 (Rognes et al., 2016). Cutadapt v1.11 (Martin, 2011) was used for removal of adapters and primers. Reads smaller than 100 bp were trimmed with vsearch, followed by dereplication. Singletons and chimeras were filtered using the UPASE pipeline (Edgar, 2013), and the reads were clustered to operational taxonomic units (OTUs) with the command – cluster_otus. Reads were mapped back (including singletons) to the filtered clusters with 99% similarity using usearch v9.0.2132 (Edgar, 2010) in order to create the OTU table, then subjected to the post-clustering algorithm LULU (Frøslev et al., 2017) as implemented in R v3.4.1. Filtered OTUs were then aligned with the reference UNITE+INSD database released on 2017.12.01 (UNITE Community 2017) for taxonomic assignment to genus level, with 97% identity threshold, 70% coverage in BLAST using QIIME v1.9.1 (Caporaso et al., 2010) with a modified assign_taxonomy.py script. OTUs that did not obtain taxonomic assignment in the above were labeled as “No blast hit.”

Metabarcoding data were analyzed using *phyloseq* (McMurdie and Holmes, 2013) framework in R (v3.4.0). OTUs with fewer than 10 reads (Werner et al., 2012; Oliver et al., 2015) and samples with fewer than 1000 reads were discarded for further analyses. Furthermore, OTUs with “No Blast Hit” were also removed from analyses. The α-diversity was evaluated with relative abundances and Hill numbers (Hill, 1973). Hill numbers were calculated using *q*-values between 0 and 3 with 0.001 intervals (Alberdi and Gilbert, 2019). The resulting matrix was decomposed with principal components analysis (PCA), with retention of the first principal component as a measure of the overall effect of α-diversity. The variance of the Hill numbers was additionally retained for visualization. Heatmap visualization and clustering were done on variance stabilized transformed (Anders and Huber, 2010) count data using *DESeq2* (Love et al., 2014) with hierarchical clustering using weighted linkage (WPGMA) (Sokal and Michener, 1958) on Pearson correlation. Furthermore, differential abundances and corresponding log2 fold changes and adjusted *p*-values were found using *DESeq2* (Love et al., 2014). *DESeq* function parameters were set as following: test type: “Wald”; fittype: “parametric.” Significant differences between relative abundances were controlled by setting false discovery rate (FDR) at 5% using the method by Benjamini and Hochberg (1995). OTUs whose abundances differed significantly between vineyards were visualized with letter-value boxplots (boxenplots) (Hofmann et al., 2017) of the raw count data.

#### Metagenomic Data Analyses

The metagenomic data analysis consisted of two stages. In the first stage, the raw sequence reads were analyzed individually for each sample. In the second stage, a binning approach was used, where analyses were performed on the total sequence data from each of the two vineyards (specifically vineyard 4 and vineyard 5). This was used to specifically explore for differences in the microbial communities relating to alcoholic fermentation or vineyard of origin.

For all analyses, the qualities of all paired-end reads were checked using FastQC (Andrews, 2010), both before and after adapter removal using Trim Galore (Krueger, 2012) and Cutadapt (Martin, 2011), with the following parameters: default Phred score: 20 and cutoff for read length: 40 bp for filtering. All reads containing “N” were also filtered and the filtered reads were subsequently merged to compile with IDBA-UD (Peng et al., 2012) for sequence assembly. MEGAHIT v1.0.4 (Li et al., 2016) was then used for sequence assembly, with metagenomic parametrization for either individual samples (Stage 1) or binning by individual vineyards (Stage 2).

For taxonomic assignment of the trimmed metagenomic reads, a curated database was constructed using the genomes of 130 relevant species, including eukaryotes and bacteria. This database is similar to, but expanded on, that used in a previous study (Sternes et al., 2017) (Supplementary Table S4B). These sequences were obtained from NCBI and used to construct a custom Kraken database. Relative abundances were obtained by mapping using Kraken v.1.0 (Wood and Salzberg, 2014) and Braken (Lu et al., 2017). Further data analysis was performed in R v.3.4.4 with *phyloseq* (McMurdie and Holmes, 2013) and other custom scripts. The Kraken mapped reads that were higher than 0.00001 relative proportion were retained for clustering which was done on Pearson correlation similarities using affinity propagation (Frey and Dueck, 2007; Bodenhofer et al., 2011). A general workflow to assess the most suitable number of clusters was started by setting the exemplar preferences value high, which led to a very large number of clusters. Application of agglomerative clustering on the resulting affinity propagation clusters allowed an inspection of the corresponding dendrogram. Based on the dendrogram, a cutoff was manually decided and affinity propagation was rerun repeatedly to achieve the desirable number of clusters. Prodigal (Hyatt et al., 2012) was used to predict the open reading frames (ORFs), with parameterization for metagenomes of individual samples (Stage 1), or binned by samples per vineyard (Stage 2), as detailed above. In order to perform the functional analysis, the ORFs were then used as an input for eggNOG (Powell et al., 2014), to obtain KO (KEGG Orthology) assignments and Clusters of Orthologous Groups (COGs) as functional annotations. The unique KOs generated for each sample individually were combined using custom Python and R scripts and were used to create a matrix of total 8744 KO, which were combined in a matrix with unique 412 pathways according to KEGG for all samples together. The KO pathway enrichment analysis was done using the following packages: Biostrings (Pagès et al., 2017), ggplot2 (Wickham, 2016), reshape (Wickham, 2007), KEGGREST (Tenenbaum, 2018), lattice (Sarkar, 2008), apcluster (Frey and Dueck, 2007; Bodenhofer et al., 2011), BioServices (Cokelaer et al., 2013), and pandas (McKinney, 2011).

For the binning approach (Stage 2), assemblies were performed with MEGAHIT on the pooled data from vineyards 4 and 5, respectively, in order to create their corresponding contig files. Next, the trimmed sequences from the data pre-processing steps for each sample were mapped to their corresponding vineyard assembled contigs files using the bwa-mem algorithm v0.7.15 (Li, 2013). The mapped sequences were subsequently cleaned of PCR duplicates using samtool v1.6 (Li et al., 2009) and exported as BAM files for binning. Binning was subsequently performed and the output was visualized with the metagenomics workflow^1^ in Anvi’o v5.1’s interactive interface (Murat Eren et al., 2015). Each assembled contig was used to create corresponding Anvi’o contig databases using the default settings. The databases were then run under HMMER v3.1.b2 (HMMER, 2015) for sequence searching using hidden Markov models (HMMs), and genes were annotated with functions from the NCBI’s COGs (Galperin et al., 2015) using command anvi-run-ncbi-cogs. The genes in the contig database were classified using Kaiju v1.5.0 (Menzel et al., 2016), with NCBI’s non-redundant protein database including fungi and microbial eukaryotes. Each of the sample bam files derived from a single vineyard was profiled with their corresponding annotated contigs database, with minimum contig length set to 2500 nt using the command anvi-profile, then merged to generate bins using CONCOCT as implemented in Anvi’o. Bins with completeness ≥40% and redundancy ≤10% were retained for analysis (Delmont et al., 2015). Abundance of the reads was estimated in terms of percentage of read recruitment. This was calculated by the mean coverage of each split in each bin with normalization of all bins respect to each other in each sample. For functional assignment of the bins, 6394 KOs were generated when combining the 11 bins, which resulted in 411 unique pathways.

All figures, except those from Anvi’o were visualized using ggplot2 (Wickham, 2016), Matplotlib (Hunter, 2007), and Seaborn (Waskom et al., 2017), with further post-processing done in Inkscape v0.91^2^. The sequencing data were deposited to European Nucleotide Archive under study number: PRJEB30801 and ERS3017411-ERS3017414, ERS3017423-26, ERS3017435-38, ERS3017447-50, ERS3017459-62, ERS3017471-74, ERS3017483-86, ERS3017495-98 in study number: PRJEB29796.

**FIGURE 1:**
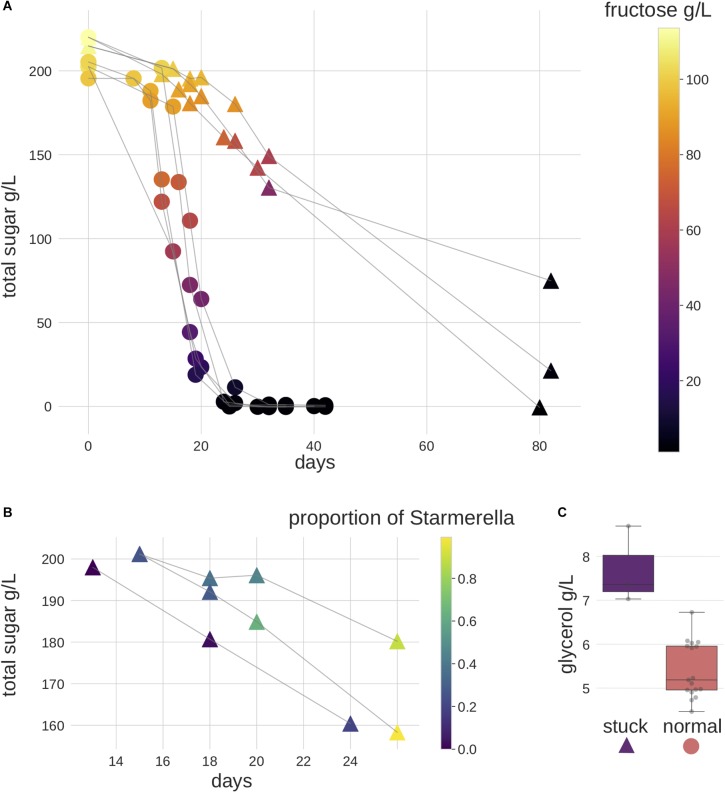
Fermentation performance of the samples. Triangles represent stuck behavior samples and circles represent normal behavior samples. **(A)** Most samples showed normal alcoholic fermentation, three samples stood out with a slower fermentation speed, and showed a preference for fructose instead of glucose. Color scale represents fructose (g/L). **(B)** A zoom in of stuck behavior samples between fermentation days 13–26. *Starmerella* was found to be the highest in relative abundance in these samples in ITS2 metabarcoding data. Color scale represents the proportion of *Starmerella*. **(C)** The glycerol concentration was higher in these stuck behavior samples when less than 20 g/L of fructose remained. The gray dots represent the actual measured values.

## Results

Eight different Riesling musts, containing four vineyard specific microbial compositions, were allowed to ferment spontaneously. While most of the samples underwent alcoholic fermentation following the expected timeline (Supplementary Table S1), three exhibited sluggish (henceforth referred to as “stuck”) fermentation behavior. Specifically, these needed 6 months (data not shown) to finish the fermentation, as opposed to the 5 weeks time needed by the others (Figure 1A). Furthermore, no malolactic fermentation was observed (Supplementary Table S1).

Metabarcoding was performed by amplifying the ITS2 gene for the 32 extracts (four sampling dates for the eight samples). Prior to subsequent analysis, one extract (sample w4b112 representing the stuck fermentation behavior group) was removed due to yielding fewer than 1000 reads. In total, 2.79 million reads were generated yielding 105 OTUs, although after all filtering this was reduced to 2.75 millions reads representing 72 OTUs that were retained for subsequent analyses (Supplementary Table S5).

A total of 1.6 billion raw reads was generated from the 10 shotgun metagenomic sequenced samples, of which 1.5 billion were retained after adapter removal. Details of the number of reads per sample and the percentage that mapped to the curated database are shown in Supplementary Table S4.

**FIGURE 2:**
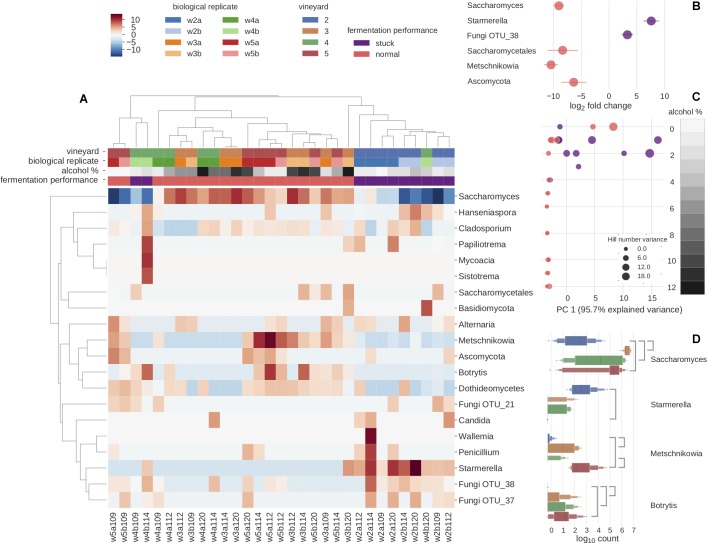
Microbial diversity and predominant genera explain the sample differences using ITS2 metabarcoding. **(A)** Clustered heatmap depicting the 20 most abundant species. Clustering is mainly driven by fermentation performance. Color scale represents species relative abundances transformed to Poisson dissimilarities. **(B)** Differential abundance of OTUs in log_2_ fold change by the fermentation performance: normal fermentation (pink) and stuck fermentation (purple). Four OTUs are found to be significantly more abundantly expressed in normal fermentations, whereas two OTUs are found significantly more abundantly expressed in stuck fermentations. **(C)** The relationship of alcohol percentage and α-diversity was measured as a first principal component of decomposed Hill numbers with *q*-values between 0 and 3, for all samples is found to decrease and vary more with rising alcohol percentage. The size of dots increases with the increase in Hill’s number variance. The gray scale corresponds to alcohol percentage. **(D)** Vineyards were studied with pairwise comparisons of differentially expressed abundances across all time points. Line between vineyards indicates a significant difference between the pairwise comparison.

### Overall Community Differences

Overall, while the metabarcoding results clearly show that *Saccharomyces* drives the alcoholic fermentation (given their abundance among the data), we observed three main drivers of sample clustering that relate to the microbial composition. First, there were differences in fermentation behaviors; second, there were differences between the four vineyards; and third there were clear differences relating to the stage of fermentation as expressed as alcohol percentage (Figure 2). Given these results, we therefore explored three principal questions. First, we used our metabarcoding and metagenomic data to explore how the microbial communities vary between vineyards. Second, we investigated how different stages within alcoholic fermentation impact the microbial biodiversity. This was done using both the metabarcoding data from all the samples, as well as the metagenomic data generated from a subset of samples chosen so as to give more detailed preliminary insights into the longitudinal effect at two of the vineyards. Third, we used the metabarcoding data to investigate how the microbial community profile differs between the normal and stuck fermentations.

**FIGURE 3:**
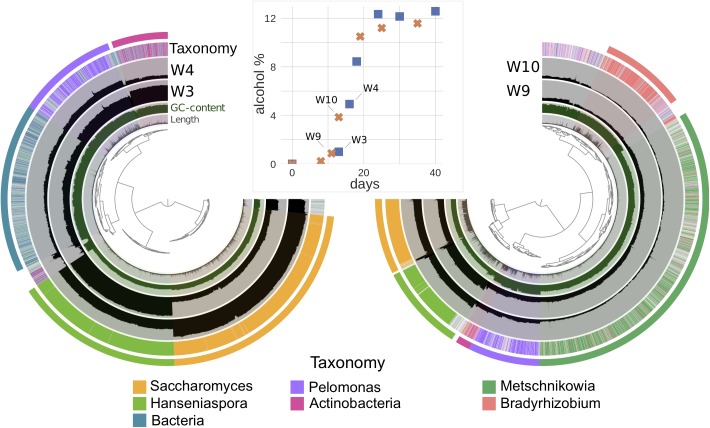
The visualization of mean coverage of genome bins in samples W3 and W4 in vineyards 4 (left), and W9, W10 in vineyard 5 (right) using Anvi’o and their corresponding alcohol percentages during fermentation (middle; blue square: vineyard 4; orange cross: vineyard 5). GC-content, length of each contig, and taxonomy classified by Kaiju were displayed in adjunct layers. Each sample is represented in a separate layer, and each bar inside the sample layer corresponds to a datum computed for a given split, where contigs longer than 20k bp were divided to different splits. The outermost layer was the bin layer, where corresponding colors linked with the taxonomy of each bin. Vineyard 4 with 4333 contigs (minimum length: 2500 nucleotides, total nucleotides: 27.29 Mbp) that represented 4% of all contigs and 36% of all nucleotides found in the vineyard 4 contigs database (93,546 contigs and total nucleotides: 74.84 Mbp). Vineyard 5 with 5364 contigs (minimum length: 2500 nucleotides, total nucleotides: 53.01 Mbp) that represented 5% of all contigs and 50% of all nucleotides found in the vineyard 5 contigs database (92,948 contigs and total nucleotides: 105.44 Mb). The hierarchical clustering of contigs based on the sequence composition and their sample distribution were used for the dendrograms at the center of Anvi’o visualization. More bin details are shown in Supplementary Table S6.

### Differences Between Vineyards

The differences between the four vineyards were initially investigated by metabarcoding all samples for the ITS2 region (Figure 2). Pairwise comparisons of the vineyards indicated that the microbial community of vineyard 2 diverged most from the three other vineyards, with the differential abundances of four fungal OTUs clearly standing out (Figure 2D). First, *Botrytis* (3: *p-adj* = 3.51e-7, 4: *p-adj* = 1.68e-3, 5: *p-adj* = 1.76e-5) and *Saccharomyces* (3: *p-adj* = 4.00e-8, 4: *p-adj* = 1.78e-4, 5: *p-adj* = 5.81e-5) were found to be significantly lower in vineyard 2. Besides the lower abundance of *Saccharomyces*, the vineyard 2 was also showcased a slower fermentation rate (Supplementary Tables S1, S5). Second, *Starmerella* was found at higher abundance in vineyard 2 than vineyard 5 (*p-adj* = 4.21e-06). Lastly, *Metschnikowia* was found to be significantly less abundant in vineyard 2 than vineyard 5 (*p-adj* = 1.39e-09) and vineyard 3 (*p-adj* = 7.56e-05). Thus, vineyard 2 was found to be the most different as it differentiated from the others with 3 known fermenting yeast genera and the grape bunch rot (Figure 2D). The only significant difference was found when comparing the other vineyards was between vineyards 4 and 5, where one OTU, *Metschnikowia* (*p-adj* = 3.50e-4), was found at significantly higher concentrations in vineyard 5 (Figure 2D). Since the differences among vineyards 3, 4, and 5 were minimal in metabarcoding, in order to investigate further, we chose a subset of samples from these three vineyards for a further metagenomic analysis. We were able to further explore this specific difference of *Metschnikowia* between vineyard 4 and vineyard 5 using the metagenomic data derived from samples from vineyards 4 (W3 and W4) and 5 (W9 and W10) that were taken at similar fermentation stages as estimated by alcohol percentage (Figure 3 and Supplementary Table S2). Annotating reads to the curated taxonomy database showed that samples with lower alcohol percentage had a lower percentage of mapped reads (W3: 15.33% and W9: 34.98%) compared to those with higher alcohol percentage (W4: 77.69% and W10: 50.54%) (Supplementary Table S4A). This suggested that the curated database was performing better in annotating higher alcohol percentage samples, thus, failing to catch species in the start of the fermentation. We reasoned that a gradual database curation alone would not cure the low annotation problem. Therefore, we resorted to functional annotation and binning-based approaches, which complement the raw reads recruitment approach as they do not rely on the same database.

Next, a functional comparison of the microbes at these two vineyards was performed using the COG classification derived from eggNOG annotation. The count number of bacterial functions was significantly higher in vineyard 4, whereas the vineyard 5 had a small increase of Saccharomycetaceae functional count and a significant increase of the “other fungus” functional count (Supplementary Figure S1). In order to further explore this non-Saccharomycetaceae group, and to validate if this corresponded to the presence of *Metschnikowia* species (as observed with metabarcoding), binning was applied with the aim of reconstruction of draft genomes.

Figure 3 shows an overview of the binning results, and the comparison of the differences between vineyards 4 and 5 corresponding with regards to alcohol levels. From vineyard 4, five genomic bins were obtained by assembly and binning. These varied in size between 1.87 and 9.78 Mbp, with completeness varying from 44.8–94.96%, and redundancy in 0–10% (Supplementary Table S6). For vineyard 5, six genomic bins were found. These varied in sizes between 2.34 and 12.3 Mbp, with completeness ranging 54.22–97.84% and redundancy in 0.72–9.35% (Supplementary Table S6). These vineyards had two yeast and two bacterial bins (*Hanseniaspora*, *Saccharomyces*, unresolved Actinobacteria, and *Pelomonas*) assigned to same taxa by annotating the genes to the NCBI’s non-redundant protein database. Furthermore, vineyard 4 also had a unique unresolved bacterial bin, while vineyard 5 was found to have two additional bins: one for the non-*Saccharomyces* yeast *Metschnikowia*, and another one for the bacteria *Bradyrhizobium* (Figure 3 and Supplementary Table S6).

As expected in the functional analysis, differentiation was found between fungi and bacteria in the form of two distinct clusters (Supplementary Figure S2). Moreover, for both clusters, we found a high functional similarity between the same species coming from different vineyards.

The abundance of *Hanseniaspora* reads was found to be higher in samples W3 (72.0%) and W9 (44.2%) in vineyards 4 and 5, respectively, which had alcohol levels of ca. 1%. At the next time point sampled (equivalent to around 4% alcohol level), we observed a decrease in relative abundance: W4 (8.2%) and W10 (22.8%) (Figure 3 and Supplementary Table S6). Furthermore, *Saccharomyces* drastically increased by 27-fold in vineyard 4 and threefold in vineyard 5, between the 1–4% alcohol level (Supplementary Table S6). This indicated the beginning of its early dominance in alcoholic fermentation. Of the other non-*Saccharomyces* yeasts solely found in vineyard 5 bins, *Metschnikowia* exhibited a similar trend to *Hanseniaspora*, with a reduction in its amount corresponding to the increase in alcohol level (Supplementary Table S6).

Interestingly, it was observed that a higher total number of bacterial related contigs, as well as the percentage of read recruitment, was obtained while a lower number of genes was identified in vineyard 4 than vineyard 5 (Supplementary Table S6). This might be due to a greater bacterial diversity in vineyard 4 than vineyard 5. Additionally, a decreasing trend in percentage of recruitment of all bacterial genomic bins was observed together with the increase in alcohol level in vineyard 4. This followed the observation in the previous subsection with the increase of *Saccharomyces* activity. The *Metschnikowia* bin had 97 unique KOs, with 14 of those characterized as NADH dehydrogenase belonging to oxidative phosphorylation.

**FIGURE 4:**
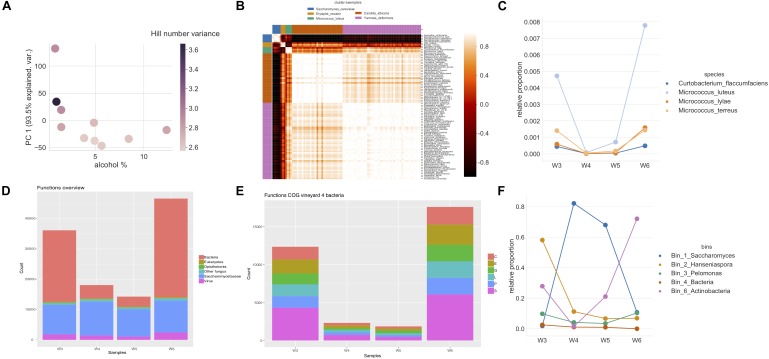
**(A)** Alcohol percentage and relationship to α-diversity of metagenomic sequenced samples of mapped reads show a decrease in α-diversity while gaining again in the end of fermentation. The α-diversity is shown as a first principal component of decomposed Hill numbers with *q*-values between 0 and 3. Color indicates change in variances of log10 fold Hill numbers. **(B)** A heatmap using pairwise clustering of mapped reads assigned to species. The assigned taxonomies generated from mapping to raw reads of the four samples from vineyard 4 to a curated database were clustered to observe patterns using affinity propagation of Pearson correlation similarities of the mapped reads assigned to species. Five clusters were found with corresponding cluster exemplars (blue: *Saccharomyces cerevisiae*, orange: *Erysiphe necator*, green: *Micrococcus luteus*, red: *Candida albicans*, purple: *Yarrowia deformans*). **(C)** Lineplots of the fifth clustered group of *Micrococcus* in **B** show that these mapped reads assigned to species decrease during alcoholic fermentation, but also start increasing again in the end. **(D)** An overview of functional analysis of different groups: bacteria, eukaryotes, opisthokonts, other fungi, Saccharomycetaceae, and viruses based on the read counts in vineyard 4 during alcoholic fermentation. Bacterial gene counts are observed to be most affected by the stage of fermentation, while other groups remain stable. **(E)** An overview of the changes in the bacterial functions to orthologous groups (COG) in vineyard 4. The COG groups are C: energy production and conversion, E: amino acid transport and metabolism, G: carbohydrate metabolism and transport, L: replication, recombination, and repair, P: inorganic ion transport and metabolism, and S: function unknown. **(F)** The relative abundance of binned read recruitment of Anvi’o results. The size of dot increases with the increase in percentage of recruitment. The *Saccharomyces* bin is observed to grow during fermentation, while the unknown Actinobacteria bin is observed to increase in the end of fermentation.

### Alcoholic Fermentation Biodiversity

We explored the effect of alcohol level on microbial biodiversity using both the metabarcoding and metagenomic datasets (See Supplementary Tables S4, S5 for details). Overall biodiversity was found to decrease during alcoholic fermentation. Specifically, the α-diversity, estimated using decomposed Hill numbers, clearly decreased with the fermentation progress (as measured in alcohol percentage) in both the metabarcoding (Figure 2C) and metagenomic datasets (Figure 4A). Interestingly, the variance of Hill numbers generated and the α-diversity was found to increase with metagenomic filtered reads (Figure 4A and Supplementary Table S4), suggesting increased species diversity. This observation was further examined by focusing the analysis on a single vineyard (vineyard 4), using the shotgun metagenomic data generated at the four different alcoholic fermentation stages (W3–W6, Figure 4 and Supplementary Table S2). The assigned taxonomies were generated by mapping reads from these samples to the curated taxonomy database used in the above subsection for metagenomic analysis (Supplementary Table S4B). In order to identify microbes that followed similar trajectories across the fermentation, affinity propagation clustering was applied on the relative abundances of species using Pearson correlation.

Five clusters were found (Figure 4B), and more in-depth investigation was shown in Supplementary Figure S3. The first contained species which showed an increase in their abundances during the fermentation (*S. cerevisiae*) (Supplementary Figure S3A). This principally implied that they were growing and active during fermentation. A cluster that included *Vitis vinifera*, *Botrytis cinerea*, and *Erysiphe necator* also showed a slight decrease at the end of alcoholic fermentation (Supplementary Figure S3B). This indicated that they were relatively stable during the fermentation progress, while the relative abundance shifts might relate to sequencing effects and cell/DNA degradation which is quite understandable for grapevine DNA. The other two grape mold species in the same cluster can be deduced as originating from the same effect based on this unsupervised clustering of Pearson correlation. A third, ambiguous, cluster showed a drastic decrease from the start, and no further increase until the end of fermentation (Supplementary Figure S3C). *Hanseniaspora* dominated in this cluster, that mainly consisted of non-*Saccharomyces* yeasts that also acted similarly (Supplementary Figure S3C). This suggested that these species were active in the early fermentation, but perished as the fermentation progressed. The fourth cluster showed a drastic decrease followed by an increase during the end of wine fermentation (Supplementary Figure S3D). This group was found to consist of both bacteria and fungi. We note that the bacteria found in this group belong to families that have been observed to relate to potential contamination originating from the commercial DNA extraction kit (Salter et al., 2014) – although others have also reported them to be found in grapes and wine (Liu et al., 2017). The relative abundances of the final (fifth) cluster that principally contained *Micrococcus* remained in some samples unchanged, and decreased in others during the initial phases of fermentation, before increasing and ending up higher than the abundances at the start of fermentation (Figure 4C).

As the fifth cluster showed unexpected behavior, further investigation was performed through functional analysis on the ORFs obtained with the metagenomic sequencing. Using the eggNOG taxonomic annotation, we separated each sample based on functions derived from fungi, bacteria, virus, opisthokonts, and other eukaryotes. The majority of the observed functions derived from fungi and bacteria (Figure 4D). A high number of functional counts that belonged to bacteria were observed at both the start and end of the fermentation; however, functional counts for fungi remained similar (Figure 4D). Therefore, the bacteria were further investigated by categorizing these genes to different COGs. The eggNOG functional classes allowed investigation of gene functional categories of selected microbial groups (Figure 4E). We observed a significant increase of bacterial functions in the first and last time points, where the last time point ended up higher than in the start of fermentation. A high portion of the genes could not be resolved and were assigned with unknown function. The read recruitment on five genomic bins yielded from assembly and binning were also investigated. The reads mapped to *Hanseniaspora* and *Saccharomyces* bins were found to alter as observed with α-diversity during fermentation. When looking into the relative abundance of recruited reads, a bin assigned to unknown Actinobacteria was observed to be increased in abundance in the last sampling date of the fermentation. This bin had 1969 genes identified with a 82.01% completeness and 0.72% redundancy [1.87 total size (Mb) and 319 contigs] (Supplementary Table S6).

In order to compare the level of fungal cells between samples, we performed qPCR targeting the ITS2 gene. Our data indicate that the overall level of fungal cells was lower in stuck samples (Supplementary Figure S4). We subsequently used the metabarcoding data to explore the microbial community differences between the stuck versus normally behaving ferments. Differential abundance analysis of the data enabled us to identify six OTUs that showed significant differences between the two phenotypes. In particular, two OTUs (a *Starmerella* species and an unknown fungal OTU) were significantly more abundant in the stuck samples (Figure 2B). The normal ferments contained a significantly higher abundance of four Ascomycete OTUs, of which three subsequently were classified to Saccharomycetales order (*Saccharomyces*, *Metschnikowia*, unknown Saccharomycetales OTU), while the fourth remained unresolved. For stuck samples, the relative abundance of *Starmerella* species was observed to increase in each sampling time relating to longitudinal direction (Figure 1B), whereas *Saccharomyces* tended to dominate the normal fermentation behavior phenotype. Chemical analyses identified several interesting observations on the stuck samples. First, the fermentation speed was slower, and all fructose was consumed before glucose. Second, the glycerol concentration was observed to be higher in these samples than in the normal performing samples, when investigating the samples with less than 20 g/L of fructose (Figure 1C). This suggests that the microbial community in the stuck samples preferably consumed fructose over glucose when the fungal amount in the community was lower.

## Discussion

Our application of metabarcoding and shotgun metagenomic sequencing techniques to spontaneous Riesling ferments from four German vineyards enabled us to shed new insights into a number of questions of relevance to winemaking. Although we find regional differences and that of biodiversity decreases throughout wine fermentation in general, such findings are observed in other amplicon sequencing-based approaches (Stefanini et al., 2016; Belda et al., 2017). Thus, we here discuss in further detail some of the other findings.

### While Metagenomic Approaches Complement Metabarcoding Data by Providing Preliminary Insights Into Functional Analysis, Some Caveats May Be Warranted

Although both metabarcoding and metagenomic approaches showed that *Metschnikowia* drove the difference between vineyards 4 and 5, caveats may be needed when interpreting the results. First, while the two sequencing methods offer broadly similar information, differences in relative abundances do exist. One key difference is similar to that previously suggested in the first shotgun sequencing paper applied to wine samples, where a *Metschnikowia* abundance bias was found between shotgun analysis and ITS2 marker gene, with ITS2 marker gene overestimating (Sternes et al., 2017). Further research and comparisons of relevant methodologies and workflows will be needed to investigate such different bias further.

Another well-known shortcoming of HTS-based approaches relates to limitations with current reference databases. It has been long acknowledged that ITS and other fungal marker gene regions have limited potential for resolving species identity (Scorzetti et al., 2002; Stefanini and Cavalieri, 2018), and metagenomic reference databases face similar challenges when assigning taxonomic labels to metagenomic DNA sequences. For example, we found that several *Saccharomyces* species besides *S. cerevisiae* were mapped in our data, namely, *Saccharomyces paradoxus* and *Saccharomyces pastorianus* (Supplementary Table S4B). Since previous studies have reported that *S. paradoxus* is rarely found in wine fermentations (Sicard and Legras, 2011; Knight et al., 2018), and there is a close evolutionary relationship between *S. cerevisiae* and *S. paradoxus* (Borneman and Pretorius, 2015), it is possible that our mapping to *S. paradoxus* is an artifact driven by close sequence homology (Naumova et al., 2005). In order to overcome the database problems, we applied functional analysis and binning strategies to metagenomic data, to make the results less dependent on only a single database. Although our preliminary metagenomic data have small sample sizes, further studies could benefit by implementing multiple approaches.

Furthermore, for wine studies, multiple challenges exist as most of the metagenomic algorithms and annotations are built for prokaryotes, and some included archaea instead of eukaryotic taxa (West et al., 2018). We see this particularly with the assembly of overlapping reads into continuous or semi-continuous genome fragments and the results from the completeness of binning. Additionally, the taxonomic assignments to genus level are not particularly satisfying with regard to obtaining useful insights into the fermentation process, thus, resolution to species or even strain level is needed. Studies on bacterial communities have shown this is possible in theory, although challenging (Papudeshi et al., 2017; Segata, 2018). For yeasts and other eukaryotes, more work is particularly needed, as multiple species or strains may be included in a contig due to current challenges in distinguishing between related community members in both the assembly and binning processes (Imelfort et al., 2014; Luo et al., 2015; Parks et al., 2017).

Although by complementing our metabarcoding data with metagenomic information we were also able to provide preliminary functional insights, such analyses are also not devoid of their challenges. This is predominantly due to the relatively poor state of annotation for most bacterial and fungal genomes. Thus, while the potential benefits of (meta)genomic analysis are clear, inference is still based on a rather shaky foundation. We observe this with the *Metschnikowia* bin, which was found to have multiple unique functions for NADH dehydrogenases which were mapped to human diseases in KEGG, due to its oxidative phosphorylation (Schägger and Ohm, 1995). However, these functions for NADH dehydrogenase relate to iron metabolism/electron transport/respiration, which have been suggested to be up-regulated by the MarR-like protein PchR in the pulcherriminic acid biosynthetic pathway of *Bacillus subtilis* (Randazzo et al., 2016). Similarly to *B. subtilis*, *M. pulcherrima* is known to synthesize pulcherriminic acid by utilizing the iron in the growth medium and causing antimicrobial activities (Randazzo et al., 2016; Gore-Lloyd et al., 2018). This ability is suggested to be the main reason for the role of *M. pulcherrima* as a biocontrol agent against other non-*Saccharomyces* in wine (Oro et al., 2014). Additionally, this antagonistic behavior has been found to extend to bacteria in a study using culturing (Sipiczki, 2006). In our study, a higher number of bacterial genes, although a lower total number of bacterial related contigs, were identified in vineyard 5 compared to vineyard 4 (Supplementary Table S6). This could help to create a less diverse environment for *Saccharomyces* to dominate and complete the alcoholic fermentation easier (Boynton and Greig, 2016). Therefore, although our metagenomic analysis is preliminary and clearly limited by the small sample sizes, we believe they provide evidence that there will be a value in incorporating such approaches to complement metabarcoding and help advance wine fermentation knowledge.

### Although Microbial Biodiversity During Alcoholic Fermentation Generally Decreases, Metagenomic Sequencing Reveals That Actinobacteria Increase in Relative Abundance

Although we observed in both our metabarcoding and metagenomic data a decline in microbial biodiversity during alcoholic fermentation, one previous metabarcoding study reported that some bacteria become relatively more abundant (in reads count) in the later stages of fermentations (Bokulich et al., 2015). In general, lactic acid bacteria and acetic acid bacteria are well known to be able to thrive after the alcoholic fermentation. Consistent with this, we noticed in our metagenomic data from vineyard 4 an increase in reads relating to the class Actinobacteria during the later fermentation stages, both when recruiting the read results to binned data, and in the functional assignments (Figure 3, 4). Mapping of the raw reads to the curated taxonomy database tentatively suggested these have a relationship with the *Micrococcus* genus. Bacteria from the class Actinobacteria have been previously observed in wines, in particular Rieslings (Piao et al., 2015) although without further interpretation (Bokulich et al., 2013; Marzano et al., 2016; Portillo and Mas, 2016), and *Micrococcus* has been previously isolated from beer (Priest, 1999), cheese (Fontana et al., 2010), and other fermented foods where it is known to be able to influence the final product. This can happen through its ability to both produce and degrade biogenic amines through amino acid decarboxylases (Voigt and Eitenmiller, 1977; Leuschner et al., 1998) as well as, produce volatile sulfur compounds (Bonnarme et al., 2000). However, we emphasize that we were not able to map the binned data to a more detailed taxonomic level, thus are only confident on the classification of this particular bin to the class level.

The dynamic changes of the community composition during fermentation could affect the detection threshold of the above Actinobacteria, which plays a key role in the unique functional counts. It is widely known that *Saccharomyces* dominate the microbial community during alcoholic fermentation, and that the total microbial biodiversity decreases. These two patterns could be driven by two possible explanations. First, due to the proportional increase of *Saccharomyces* to other microbes – given that we maintained a relatively constant sequencing depth across the samples, we may well be missing less abundant microbes (such as bacteria). As a result, a big decline in abundance and identified functions of bacteria would be observed when *Saccharomyces* levels are at their peak (Figure 4F). A second explanation could be that the observed decline in bacteria is the actual biological behavior, for example, if bacteria enter a survival mode with drastically decreased activity alongside the rapid growth of *Saccharomyces*. Toward the end of alcoholic fermentation, the activity of *Saccharomyces* slows down and provides space for the growth of bacteria. This possibility is supported by the observed functions of other groups, such as opisthokonts, and other eukaryotes, where no clear decline is observed but rather a steady identification rate of their functions across the fermentation (Figure 4D). Additionally, we cannot rule out the phenomenon of spurious correlation that is a well-known potential occurrence in compositional data, therefore with bigger sample sizes, a more in-depth analysis to the pairwise associations could be applied. Yet, we cannot rule out that the potential functionality and the change in abundance of the actual species is a real observation, although we could not relate this to the specificities in bacterial potential functionality, as most of the annotations remained unresolved (Figure 4E). The behavior was unlike the one observed for the cluster containing *V. vinifera*. Clearly, further validation through multi-omic studies with larger sets of samples would be useful for exploring this further.

### Starmerella and Stuck Fermentation Behavior

Both our metabarcoding and chemical analyses also showed that the samples with stuck fermentation behavior during alcoholic fermentation associated with both the presence of fructophilic *Starmerella* and absence of *Saccharomyces* (Figure 1 and Supplementary Figure S4). These findings are consistent with those from previous studies in which *Starmerella* (synonym *Candida zemplinina*) had been investigated because of its known fructophilic characters, aroma profile as well as lower ethanol production, and elevated glycerol contents (Giaramida et al., 2013; Ciani and Comitini, 2015; Masneuf-Pomarede et al., 2015). Based on inoculation studies, it has been shown that *Starmerella* has a reduced rate of fermentation (Aponte and Blaiotta, 2016) and requires *S. cerevisiae* to finish the alcoholic fermentation (Masneuf-Pomarede et al., 2015). Indeed, the lower amount of fungal cells found in the samples instead of the abundant *Starmerella* might better explain the sluggish fermentation rate (Supplementary Figure S4). In addition to providing the first HTS-based insights into this, our results provide the first evidence of these characteristics in samples taken from natural winemaking environments. Our dataset also enabled us to observe that samples with more *Starmerella* had reduced levels of *Botrytis*. This is intriguing given that *Starmerella* was isolated for the first time from sweet botrytized wines with high fructose-glucose ratio (Sipiczki, 2004; Csoma and Sipiczki, 2008). Thus, we suggest that further investigation into the relationships between *Starmerella* and other fungal genera using HTS techniques may be of interest, as specific links between *Starmerella* and winemaking environment have not currently been established (Masneuf-Pomarede et al., 2015).

## Conclusion

In summary, we demonstrate the power of HTS-based tools in characterizing microbial community differences and fermentation population dynamics. For instance, we found that *Metschnikowia* drove the difference between two vineyards during alcoholic fermentation, we revealed the increase of Actinobacteria relative abundance, and that the stuck fermentation behavior during alcoholic fermentation was associated with both the presence of *Starmerella* and absence of *Saccharomyces*. While both metagenomic and metabarcoding approaches were found to deliver similar results, the former provides a more in-depth understanding given it offers an untargeted taxonomical analysis, as well as enabling insights at the functional level. Ultimately as other such studies appear, we anticipate that the HTS-based tools will catalyze significant further future wine microbial research.

## Data Availability

The sequencing data were deposited to European Nucleotide Archive under study number: PRJEB30801 and ERS3017411-ERS3017414, ERS3017423-26, ERS3017435-38, ERS3017447-50, ERS3017459-62, ERS3017471-74, ERS3017483-86, ERS3017495-98 in study number: PRJEB29796.

## Author Contributions

KS and SM conceived the ideas, designed the experiments, co-wrote the manuscript with inputs from all authors, and performed the analyses and interpretations of metabarcoding data. KS performed the sample preparation and fermentation trial set up and data collection. SM carried out the laboratory work and data collection. CM led the analysis and interpretation of metagenomic data with the inputs from KS and SM. KS and CM curated the wine metagenomic database. CC developed the shotgun library construction protocols and performed the protocol comparison with SSTM. JS, DM, UF, and MG oversaw the study and edited the manuscript. All authors read and approved the final version of the manuscript.

## Conflict of Interest Statement

JS was employed by company Chr. Hansen A/S. The remaining authors declare that the research was conducted in the absence of any commercial or financial relationships that could be construed as a potential conflict of interest.

## Footnotes

1 http://merenlab.org

2 https://inkscape.org/

## Supplementary Material

FILE S1 Shotgun library construction method comparison.

FIGURE S1 Absolute counts of functions with taxonomy separation from eggNOG results with the top 6 groups included: bacteria, eukaryotes (without fungi), opisthokonts, other fungus, family Saccharomycetaceae, and virus. Functions compared between vineyard 4 (W3 and W4) and vineyard 5 (W9 and W10) show abundances and differences caused mainly by two groups: bacteria and a collective group of “other fungus and Saccharomycetaceae.”

FIGURE S2 Heatmap visualization of the drafted genomic bins in vineyard 4 (v4) and vineyard 5 (v5) assigned with unique 6394 KEGG Orthology annotated functions using affinity propagation with Pearson correlation on the presence and absence table. Cluster colors are based on unsupervised clustering from affinity propagation.

FIGURE S3 Affinity propagation clustering of mapped taxa using Pearson correlation in metagenomic data of vineyard 4. **(A)** The species which had an increase of their abundance during the fermentation were grouped to cluster 1. **(B)** Second cluster included *Vitis vinifera*, *Erysiphe necator*, and *Botrytis cinerea*. **(C)** Third cluster was wine related yeasts driven cluster dominated with *Hanseniaspora uvarum*. The cluster shows a decrease from the start and no further increase until the end. **(D)** Fourth ambiguous cluster with yeast and bacteria.

FIGURE S4 Metabarcoding performance. Sample read numbers from metabarcoding mapped against ITS2 gene copy numbers with samples colored and shaped according to fermentation behavior. The data show that samples with stuck fermentation behavior had the lowest amounts of both reads and ITS2 gene copy numbers. Blue triangle: stuck fermentation behavior, orange circle: normal fermentation behavior.

FIGURE S5 Mean C_t_ values (±standard deviation) from qPCR of three extraction methods in different sample types. *n* = 5 for ferment, while *n* = 2 for other. Colors correspond to the three methods: dark gray: BEST, light gray: BEMT, gray: NEBNext. Refer to Supplementary File S1 for more details.

TABLE S1 Details of samples and measured wine parameters.

TABLE S2 Summary of selected 10 samples used in metagenomic sequencing.

TABLE S3 BGI 2.0 adapters sequences.

TABLE S4 **(A)** Summary of metagenomic sequencing data generated. The classified reads and unclassified reads were mapped to a curated database using Kraken and Bracken. **(B)** The relative abundance of number of mapped reads to each taxon in curated database per sample found in at least once.

TABLE S5 An overview of OTUs assignment per sample using ITS2 metabarcoding.

TABLE S6 **(A)** Summary of binning details and **(B)** percentage of recruitment for the metagenomic data of vineyards 4 and 5. Percentage of recruitment summarizes the mean coverage of each split in each bin, and normalize every bin with respect to each other. It is critical to remember that these values do not take the unassembled data into account.
