# Supplementary material for: Taxonomic and Functional Characterization of the Microbial Community During Spontaneous in vitro Fermentation of Riesling Must
Source: Front Microbiol. 2019 Apr 9;10:697. doi: 10.3389/fmicb.2019.00697 (PMC6465770; doi:10.3389/fmicb.2019.00697)
Supplement: FILE S1 — Shotgun library construction method comparison. [file Data_Sheet_1.pdf]

## Supplementary Material

### 1 Supplementary File 1

Comparison of the performance of shotgun library construction methods

**Sample preparation**

In order to test the efficiency of shotgun library construction of ferment samples, 3 shotgun library construction methods were compared: i) BEST, a recent published single-tube library construction method (Carøe et al., 2018); ii) a modification of BEST, Blunt End Multi Tubes protocol (BEMT); and iii) commercial kit, NEBNext (E6070). Five ferment samples were used in this study and subsequently fragmented to average size 300-400 bp by Bioruptor 300 (Diagenode, Belgium) using 6 cycles with 15 seconds on and 90 seconds off. Three identical volume aliquots of each of the 5 ferment samples (30µL, < 0.2 - 12ng) were used for this study. Library blanks (addition of EB buffer), a positive control of wolf DNA, and a wine sample were also included, the latter to test for effect of inhibitors that could be expected in the ferment samples. All libraries were constructed in parallel under laminar flow hood conditions.

**BEST**

The libraries were constructed based on the protocol of Carøe et al. (2018) with the addition of reaction enhancer and optimized enzyme concentrations following the protocols in Mak et al. (2017), and with a total initial input volume of 32µL. The whole process involved end repair, adapter ligation, fill-in and finally purification. In the adapter ligation step, 2µL of 10µM BEDC3 adapters (Carøe et al., 2018) were added to each library. Libraries were eluted in 50µL in EBT buffer (EB buffer from Qiagen with 0.01% Tween 20).

**BEMT**

This protocol is a modification of the BEST protocol, incorporating a purification immediately after the end repair step, so as to enable elevated enzyme concentrations to be used, and their subsequent efficient removal after end repair. We hypothesized that higher enzyme concentrations as well as the additional purification would enable more efficient library building on samples with high amounts of enzymatic inhibitors and/or high amounts of input DNA. An initial total input volume of 36.75µL was added to the end repair master mix, consisting of 2.5µL T4 DNA polymerase (3U/µl, NEB), 2.5µL T4 Polynucleotide kinase (PNK 10U/ul, NEB), 0.5µL dNTPs (25mM, Invitrogen), 5µl 10X T4 DNA ligase buffer (NEB) and 2.75µL reaction enhancer. The reaction was incubated at 20°C for 30 minutes, followed by a purification using Monarch® DNA Cleanup Columns (NEB). Purification was carried out using 450µL of modified PB buffer (Allentoft et al., 2015), the centrifuged at 6000 xg for 1 minute followed by 14000 xg for 1 minute in order to spin the PB buffer through the columns. The columns were subsequently washed with 800µL PE buffer and spun at 10000 xg,

followed by an additional spin at 17000 xg for 3 minutes. DNA was eluted in 33.8µL EBT buffer after an incubation for 15 minutes at 37 °C, by spinning at 17000 ×g. For the adapter ligation step, the same amount and concentration of adapter was used for each sample as described in the BEST section, and mixed thoroughly prior to the addition of ligation mix. Ligation master mix for each sample consisted of 6µL 50% PEG 4000 (Sigma), 1µL T4 DNA ligase (400U/µL, NEB), 5µL 10X T4 DNA ligase buffer (NEB) and 2.2µL reaction enhancer. The reactions were incubated at 20 °C for 30 minutes, followed by 65 °C for 10 minutes. After incubation, the fill-in step was carried out with 1.6µl Bst 2.0 Warmstart polymerase (8U/µL, NEB), 0.4µL 25 mM dNTPs, 2µL 10X Isothermal amplification buffer (NEB) and 6µL AccuGene molecular biology water (Lonza), followed by an incubation at 65°C for 15 minutes and 80°C for 15 minutes. Libraries were then purified using Monarch® DNA Cleanup Columns (NEB) as before, except using 300µL PB buffer and eluted in 50µl EBT.

**NEBNext**

Libraries were constructed using the components of a commercial kit, NEBNext DNA library prep master mix from NEB (#E6070). In the end repair step, 42.5µL of initial input volume was mixed with 2.5µL NEBnext End Repair Enzyme Mix and 5µL NEBnext ENd Repair Reaction Buffer, followed by an incubation at 20 °C for 30 minutes. Purification was as described in the BEMT protocol. DNA was eluted in 33µL EBT buffer after an incubation for 15 minutes at 37 °C, before spinning at 17000 xg. Purified DNA was subsequently mixed thoroughly with the same amount of adapter as described above, followed by 10µL 5X Quick Ligation Reaction buffer and 5µL Quick T4 DNA Ligase. The reactions were incubated at 20 °C for 30 minutes, and subsequently purified with Monarch® DNA Cleanup Columns (NEB) as described above, except using 300µL PB buffer and eluting in 42µL EBT. Fill-in master mix contained 3µL Bst DNA polymerase and 5µL Adapter fill-in reaction buffer, and was incubated at 65 °C for 20 minutes followed by 80 °C for 20 minutes.

**Quantitative PCR**

Quantitative real time PCR (qPCR) was performed for all libraries in 10µL reaction volumes, with 5µL LightCycler® 480 SYBR Green I Master (Roche), 0.25µL of 10µM forward and reverse primer mix (IS7 & IS8, (Meyer and Kircher, 2010), 1µL template of 20-fold dilution of library, and 3.75µL AccuGene molecular biology water (Lonza). qPCR blanks using EBT were included. qPCR was performed on a MX3005 qPCR machine (Agilent) with the following cycling conditions: 95°C for 5 minutes, followed by 40 cycles of 95°C for 30 seconds, 60°C for 30 seconds, and 72°C for 7 minutes. This was followed by incubation at 95°C for 1 minute, 55°C for 30 seconds, and 95°C for 30 seconds to, produce a dissociation curve. The values of cycle thresholds ($C_t$ values) provided a relative measurement of library molecules in each library.

**Results**

The qPCR results are shown in Supplementary Figure 5. Low $C_t$ values indicate a higher number of target molecules entering the qPCR reaction, thus higher efficiency of library construction. Efficiency in library building did not seem to significantly differ between ferment samples, positive

control, positive control with inhibitor spiked in, and inhibitor. The ferment libraries constructed using BEMT had the lowest mean $C_t$ values (data not shown) while NEBNext had a slightly higher $C_t$ values by ~1 cycle compared to the other two methods indicated lower efficiency. In the library blank, higher $C_t$ values were observed in BEMT and NEBnext which indicated a lower level of background noise, presumably from adapter-dimers. Due to the slightly better amplification efficiency in library molecules of ferment samples and lower risk of background noise when using BEMT, we chose this library construction method for the main study of ferment samples. However, more in depth study will be required for a thorough comparison among the three methods.

**Supplementary references**


Allentoft, M.E., Sikora, M., Sjögren, K.-G., Rasmussen, S., Rasmussen, M., Stenderup, J., et al., (2015). Population genomics of Bronze Age Eurasia. Nature 522, 167–172. https://doi.org/10.1038/nature14507

Carøe, C., Gopalakrishnan, S., Vinner, L., Mak, S.S.T., Sinding, M.H.S., Samaniego, J.A., et al., (2018). Single-tube library preparation for degraded DNA. Methods Ecol. Evol. 9, 410–419. https://doi.org/10.1111/2041-210X.12871

Mak, S.S.T., Gopalakrishnan, S., Carøe, C., Geng, C., Liu, S., Sinding, M.-H.S., et al. (2017). Comparative performance of the BGISEQ-500 vs Illumina HiSeq2500 sequencing platforms for palaeogenomic sequencing. Gigascience 6, 1–13. https://doi.org/10.1093/gigascience/gix049

Meyer, M., Kircher, M. (2010). Illumina sequencing library preparation for highly multiplexed target capture and sequencing. Cold Spring Harb. Protoc. 2010, db.prot5448. https://doi.org/10.1101/pdb.prot54488
