# Supplementary figures and images for: Taxonomic and Functional Characterization of the Microbial Community During Spontaneous in vitro Fermentation of Riesling Must
Source: Front Microbiol. 2019 Apr 9;10:697. doi: 10.3389/fmicb.2019.00697 (PMC6465770; doi:10.3389/fmicb.2019.00697)

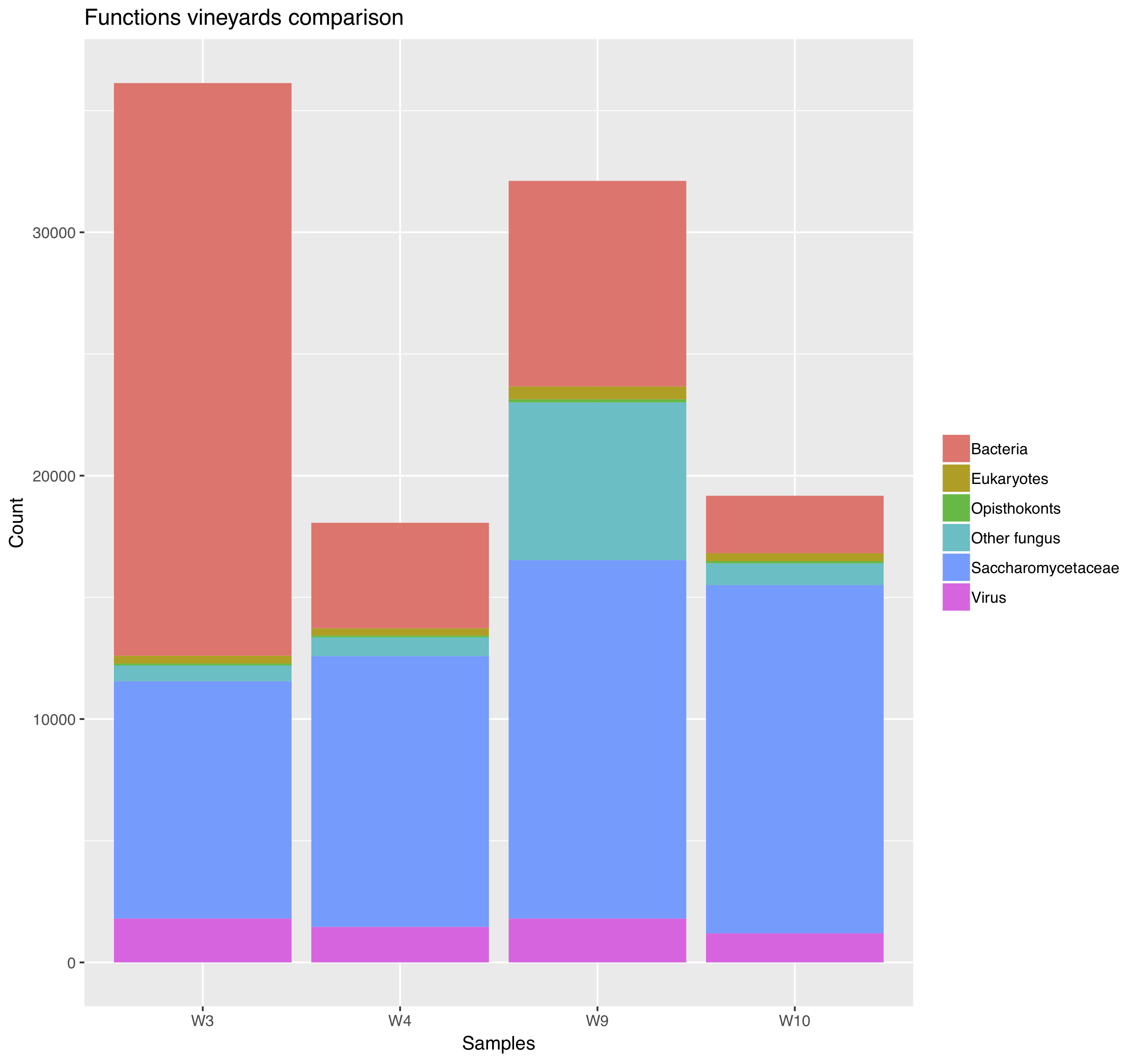

Supplement: FIGURE S1 — Absolute counts of functions with taxonomy separation from eggNOG results with the top 6 groups included: bacteria, eukaryotes (without fungi), opisthokonts, other fungus, family Saccharomycetaceae, and virus. Functions compared between vineyard 4 (W3 and W4) and vineyard 5 (W9 and W10) show abundances and differences caused mainly by two groups: bacteria and a collective group of “other fungus and Saccharomycetaceae.” [file Image_1.tiff]

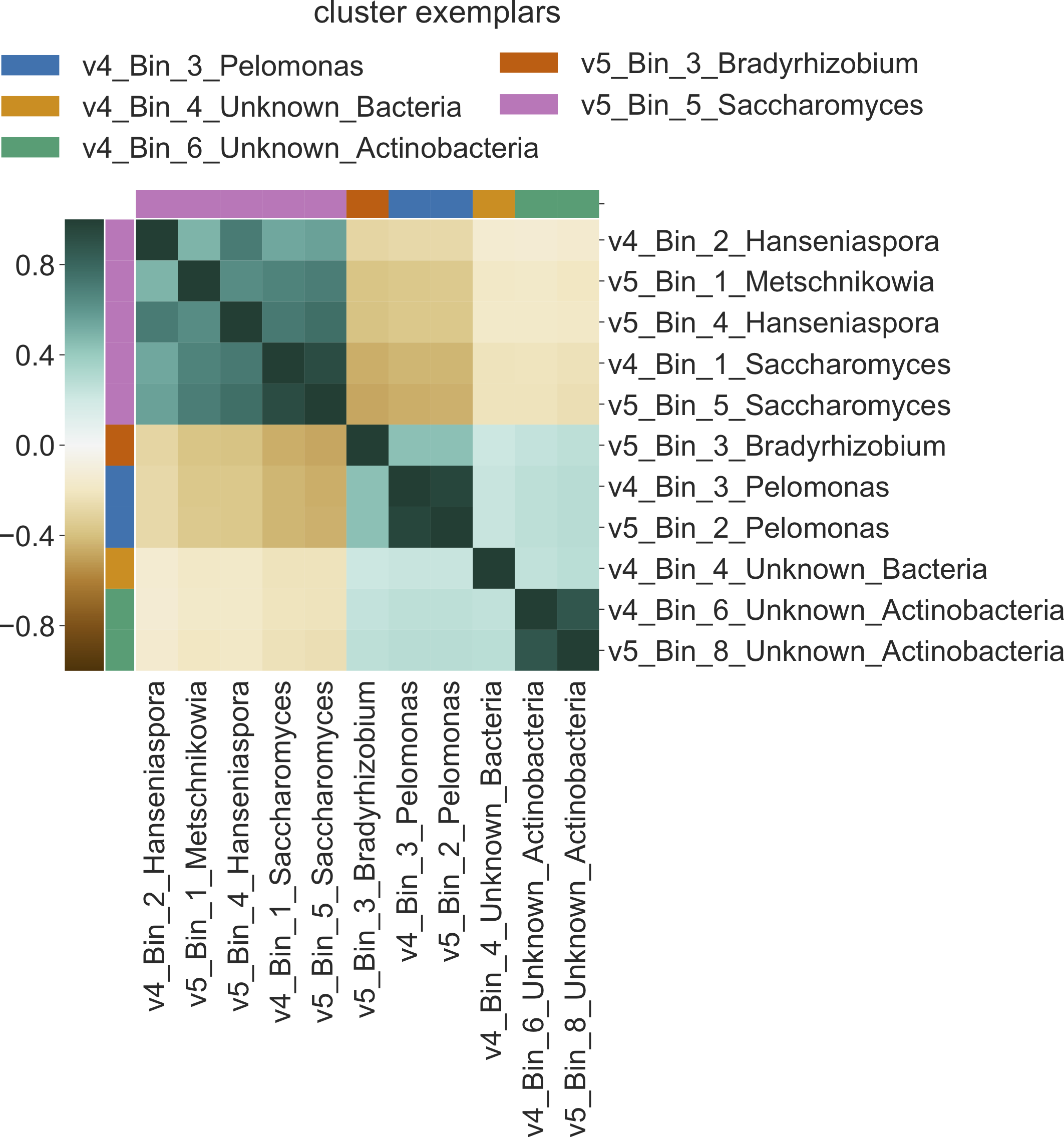

Supplement: FIGURE S2 — Heatmap visualization of the drafted genomic bins in vineyard 4 (v4) and vineyard 5 (v5) assigned with unique 6394 KEGG Orthology annotated functions using affinity propagation with Pearson correlation on the presence and absence table. Cluster colors are based on unsupervised clustering from affinity propagation. [file Image_2.TIFF]

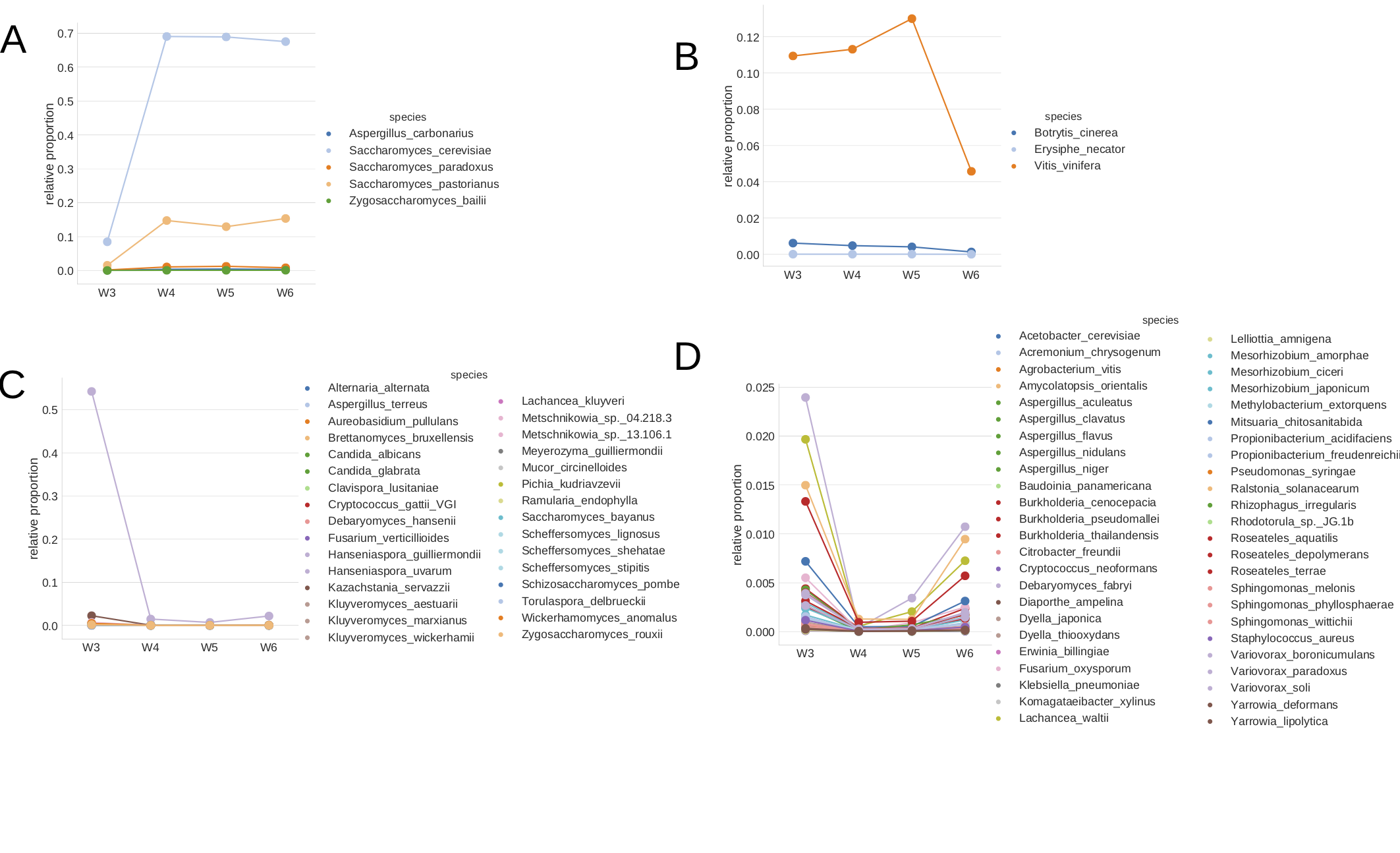

Supplement: FIGURE S3 — Affinity propagation clustering of mapped taxa using Pearson correlation in metagenomic data of vineyard 4. (A) The species which had an increase of their abundance during the fermentation were grouped to cluster 1. (B) Second cluster included Vitis vinifera, Erysiphe necator, and Botrytis cinerea. (C) Third cluster was wine related yeasts driven cluster dominated with Hanseniaspora uvarum. The cluster shows a decrease from the start and no further increase until the end. (D) Fourth ambiguous cluster with yeast and bacteria. [file Image_3.TIFF]

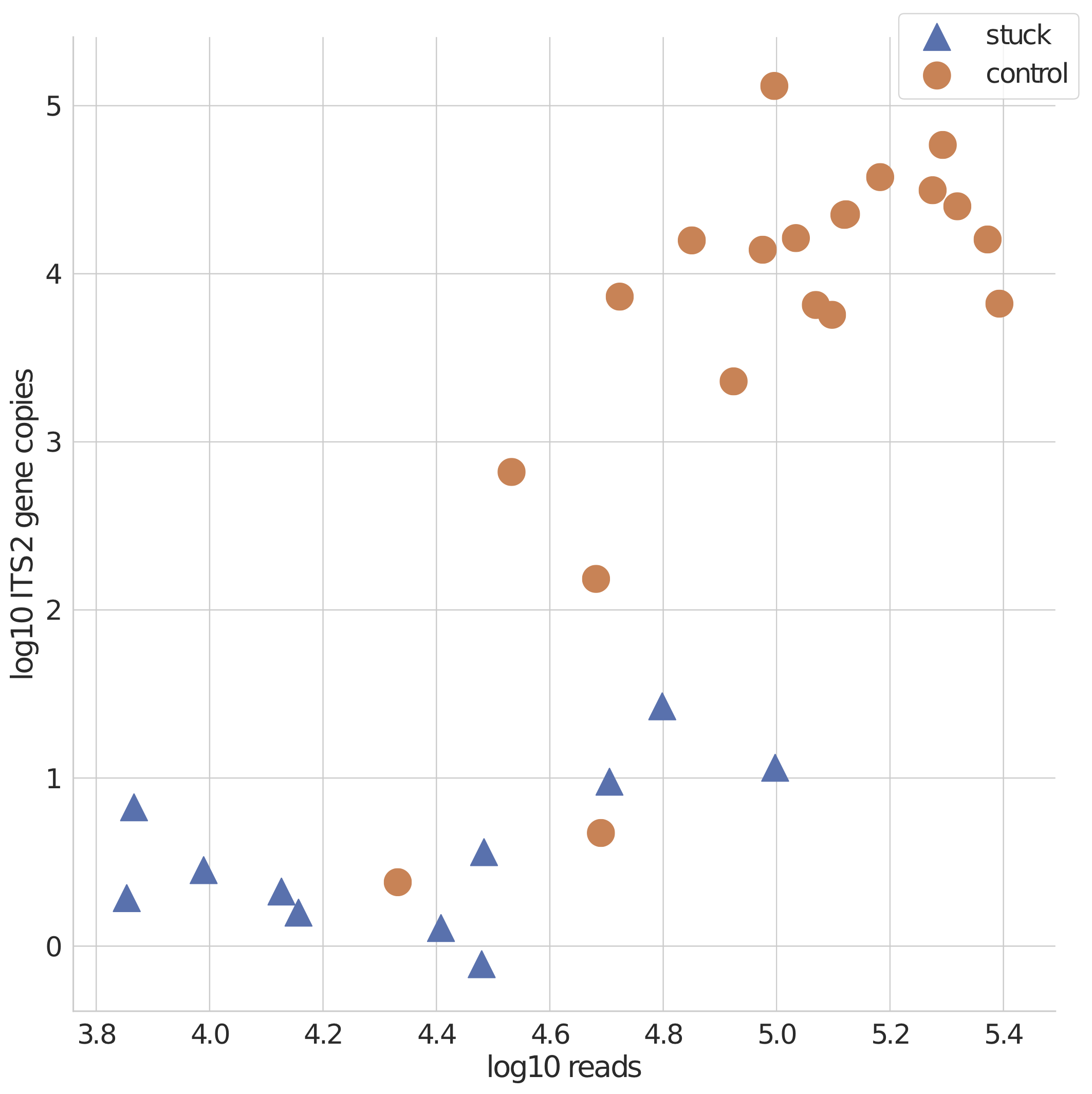

Supplement: FIGURE S4 — Metabarcoding performance. Sample read numbers from metabarcoding mapped against ITS2 gene copy numbers with samples colored and shaped according to fermentation behavior. The data show that samples with stuck fermentation behavior had the lowest amounts of both reads and ITS2 gene copy numbers. Blue triangle: stuck fermentation behavior, orange circle: normal fermentation behavior. [file Image_4.TIFF]

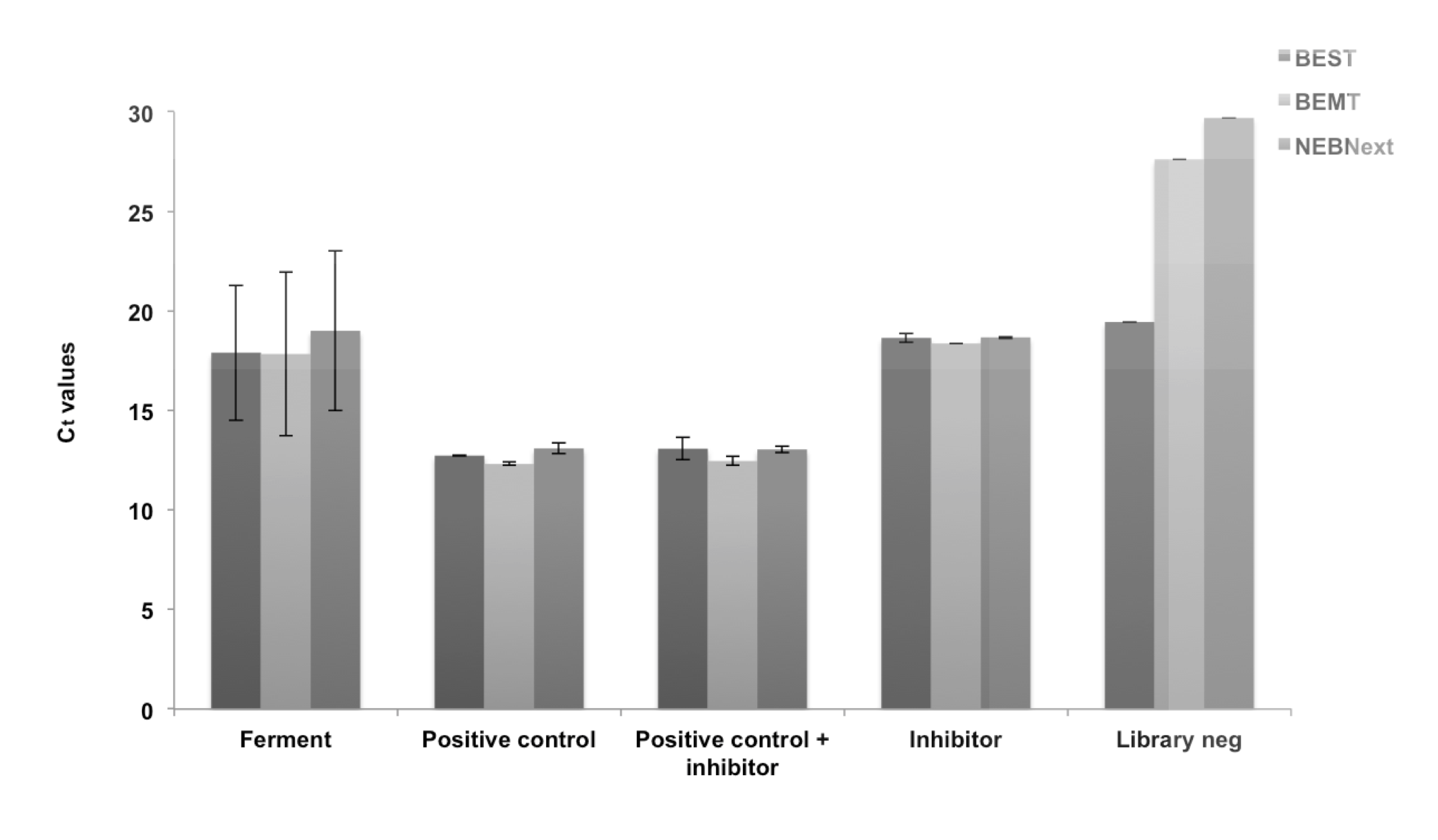

Supplement: FIGURE S5 — Mean Ct values (±standard deviation) from qPCR of three extraction methods in different sample types. n = 5 for ferment, while n = 2 for other. Colors correspond to the three methods: dark gray: BEST, light gray: BEMT, gray: NEBNext. Refer to Supplementary File S1 for more details. [file Image_5.TIFF]
